# Contribution of *Drosophila* TRPA1-Expressing Neurons to Circadian Locomotor Activity Patterns

**DOI:** 10.1371/journal.pone.0085189

**Published:** 2013-12-18

**Authors:** Youngseok Lee

**Affiliations:** Departments of Bio and Fermentation Convergence Technology, Kookmin University, Seoul, Korea; University of Houston, United States of America

## Abstract

In both vertebrates and invertebrates, Transient Receptor Potential (TRP) channels are expressed in sensory neurons and mediate environmental stimuli such as light, sound, temperature, and taste. Some of these channels, however, are expressed only in the brain and their functions remain incompletely understood. Using the *GAL4*/UAS binary system with a line in which the *GAL4* had been knocked into the *trpA1* locus in *Drosophila*, we recently reported new insights into TRPA1 localization and function, including its expression in approximately 15% of all circadian neurons. TRPA1 is expressed in lateral posterior neurons (LPNs), which are known to be highly sensitive to entrainment by temperature cycles. Here, I used the bacterial sodium channel, *NaChBac*, to examine the effects of altering the electrical properties of *trpA1* neurons on circadian rhythms. My results indicate that circadian activity of the flies in the morning, daytime, and evening was affected in a temperature-dependent manner following TRPA1 neuronal activation. Remarkably, TRPA1 neuron activation in flies kept at 18°C impacted the morning peak of circadian activity even though TRPA1 is not expressed in morning cells. Taken together, these results suggest that the activation of TRPA1-expressing neurons may differentially coordinate light/dark circadian entrainment, depending on the temperature.

## Introduction

Animals from flies to humans depend on circadian clocks to synchronize their physiology and locomotor rhythms. Circadian clocks are entrained by light and temperature changes, which vary with the Earth’s rotation. These environmental cues are directly perceived by sensory systems or clock cells in the *Drosophila* brain [1-3]. Over the previous 3 decades, there has been considerable progress towards understanding the molecular mechanisms of the *Drosophila* clock. An internal clock that counts on the circadian cycles of clock genes and proteins such as period (Per) in the pacemaker neurons controls changes in circadian activity. In standard laborary conditions, *Drosophila melanogaster* displays bimordal locomotor activity peaks at dawn and dusk. Once they are entrained by the light cycles, *Drosophila* show anticipatory behavior, which manifests itself as increased movement before the onset and offset of light. Another feature of circadian rhythm is that period length and rhythmic power are maintained in constant darkness [4].

Approximately 150 neurons in the *Drosophila* whole brain show robust oscillation consistent with clock genes such as *period* and *timeless*. These clock neurons are classified into 2 major groups based on their locations. The lateral neurons (LNs) are located in the anterior cortex between the central brain and the optic lobe, whereas the dorsal neurons (DNs) are located in the cortex of the dorsal protocerebrum (DP). These 2 groups are both further divided into 3 subgroups. The LNs include the small (s-LNv, 5 neurons) and large (l-LNv, 4 neurons) ventrolateral groups, and a dorsolateral group (LNd, 6 neurons) in each hemisphere. The DNs include the DN1, DN2, and DN3 groups, which have a proximal to distal arrangement. Another group of neurons, the lateral posterior neurons (LPNs), contains 3 pacemaker neurons in each hemisphere that show temperature entrainment [4-6]. These pacemaker neurons are categorized into morning or evening subgroups based on two peaks of daily locomotor activity [7-9]. The s-LNv are associated with morning activity, while the LNd and DN1 are associated with evening activity. These behavioral allocations are not strict and can be affected by different environmental conditions [10-12]. In addition, light sensation in the central brain requires the blue light photoreceptor cryptochrome (Cry), which functions in subsets of pacemaker neurons [1,14].

Using a *GAL4* reporter which was knocked into the *trpA1* locus, we recently showed that *trpA1* is expressed in most circadian neuron clusters, including 1 LNv, 3 LNds, 2–3 DN1s, 1 DN2, 1 DN3, and 3 LPNs [13]. We previously showed that at least 2 TRPA1-positive (*trpA1* ^+^) neurons do not overlap with the 3 Cry-positive LNds [14]. In addition, *trpA1* temperature entrainment defects driven by 29°C and 18°C can be rescued by expression of the *UAS*-*trpA1* transgene under the control of *tim*-*GAL4*, but not *cry*-*GAL4* [13]. These findings provide strong evidence in favor of the model that distinct groups of central neurons are responsible for light and temperature entrainment, and that TRPA1 selectively regulates circadian activity patterns during temperature cycles (TC). 

More recently, another TRP channel, *pyrexia* (*pyx*), has been reported to affect temperature synchronization to TCs in the relatively low temperatures (16-20°C), but not at higher temperatures (21-29°C) [15]. Pyrexia (Pyx) functions in peripeheral sensory organs. Since the threshold for the thermal activation of Pyx is ~40°C [16], these findings implicate a signaling cascade, rather than direct thermal activation of Pyx, in clock synchronization. 

Here, I further analyzed the role of *trpA1* ^+^ CNS neurons in circadian rhythms. I performed locomotor behavioral assays with light/dark (LD) cycles at 3 temperatures (18°C, 25°C, and 29°C). The analyses of hyperexcited *trpA1* ^+^ pacemaker neurons indicate other neurons outside of LNv can also control morning activity, especially at low temperatures and during daytime.

## Materials and Methods

### Fly stocks

The *trpA1* ^*GAL4*^ flies are described in a previous report [17]. The following flies were obtained from the Bloomington Stock Center: 1) *UAS-NaChBac*, 2) *w* ^1118^, and 3) *UAS*-*mCD8*::*GFP*. The *w* ^1118^ strain was used as the “wild-type” control. The *trpA1* ^*GAL4*^ and *UAS-NaChBac* strains were outcrossed at least 5 generations.

### Immunohistochemistry

Antibody stains of larval and adult brains were performed as previously described [18]. Briefly, dissected brains were placed into 24-well cell culture plates (Costar Corp.) containing 940 μL of fix buffer (0.1 M Pipes pH 6.9, 1 mM EGTA, 1% TritonX-100, 2 mM MgSO_4_, 150 mM NaCl) and 60 uL of 37% formaldehyde. The plates were placed them on ice for 45 min. Subsequently, the brains were washed 3 times (1× PBS, 0.2% saponin) and blocked for 8 h at 4°C with 1 mL of blocking buffer (1× PBS, 0.2% saponin, 5 mg/mL BSA). The brains were incubated overnight at 4°C with the primary antibodies, washed 3 times, blocked for 15 min, incubated for 4 h at 4°C with the secondary antibodies (Alexa 488 and Alexa 568; 1:200 Invitrogen-Molecular Probes), and again washed 3 times. The brain was transferred into 1.25× PDA Solution (37.5% glycerol, 187.5 mM NaCl, 62.5 mM Tris pH 8.8) and viewed by confocal microscopy (Carl Zeiss LSM510). Primary antibodies were used with the following dilutions: mouse anti-green fluorescence protein (GFP; 1:1,000 Invitrogen-Molecular Probes), rabbit anti-GFP (1:1,000 Santa Cruz), mouse anti-β-GAL (1:1,000 Promega), rabbit anti-tyrosine hydroxylase (TH; 1:200 Pel-Freez), mouse anti-PDF (1:100 Developmental Studies Hybridoma Bank), rabbit anti-Per (1:1,000), and rabbit anti-dILP2 (1:100).

### Behavioral assays

I used a *Drosophila* Activity Monitor (DAM) system and isogenic strains to examine circadian locomotor behavior. Flies that were 3–7 days old were loaded into tubes that contained 1% agarose and 5% sucrose food at one end, and then were entrained to LD cycles for 3 to 4 days before they were transferred to free running in constant dark. Data analyses were performed using ClockLab (TriKinetics) in conjunction with MatLab. Data from a 6-day span (DD2 to DD7, free running in constant dark) were examined for periodicity with a Fast Fourier Transfer (FFT) analysis. Values obtained in the FFT that were lower than 0.03 were considered arrhythmic. Anticipation index values were determined for each genotype as the largest 2-h increase in locomotor activity over the last 6-h dark phase (morning anticipation index) or 6-h light phase (evening anticipation index) with absolute locomotor activity [19]. To determine the relative daytime activity, I calculated the 6-h mid-day activity over the 12-h daytime activity. To determine the evening slope, I here calculated a ratio of the last 1 h of locomotor activity over the last 3-h light phase. To quantify the relative locomotor activity at cold phase, I determined the last 6-h cold phase over the total locomotor activity. For the statistical analysis, all data were analyzed using ANOVA and Scheffe’s post-hoc tests. A *p*-value < 0.05 was considered statistically significant.

## Results

### Expression pattern of *trpA1* in the *Drosophila* brain

**Figure 1:**
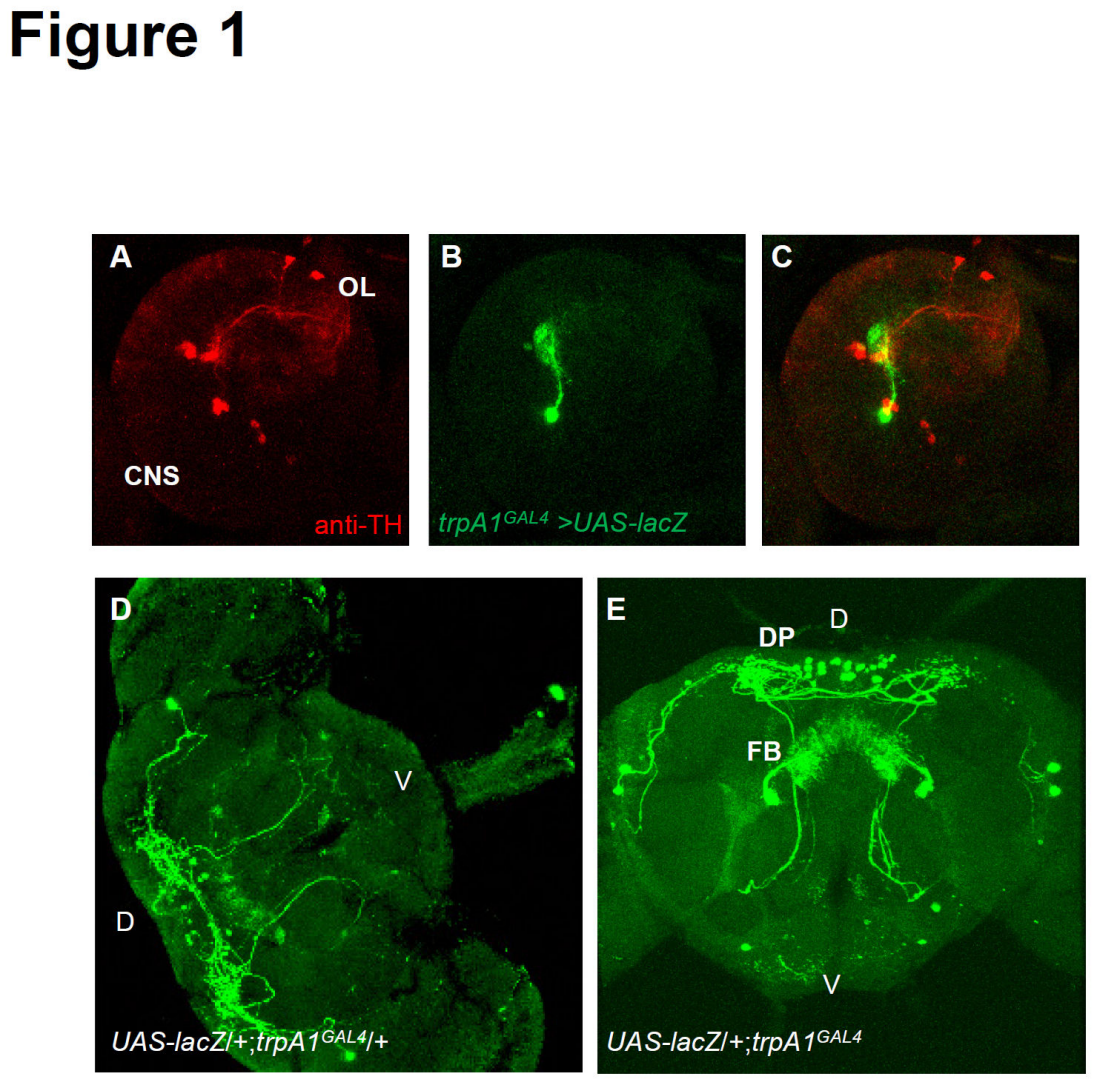
A GAL4 Knock-In into the *trpA1* locus illustrates previously unknown expression pattern of *trpA1* in the adult brain. (A–C) Brains dissected and stained with anti-TH (A) and anti-β-GAL (B) from UAS-*lacZ*/+;*trpA1* ^GAL4^/+ flies at the third instar larval stage. Merged image (C) indicates no overlap between *trpA1* reporter and dopaminergic neuronal marker. (D–E) Brains dissected and stained with anti-β-GAL from UAS-*lacZ*/+;*trpA1^GAL4^*/+ (D) and UAS-*lacZ*/+;*trpA1* ^*GAL4*^ (E) flies in the adult stage, which indicates the broad expression of the *trpA1* reporter and neural innervation of the fan-shaped body (FB) and the dorsal protocerebrum (DP). Abbreviations: D, dorsal; V, ventral.

*Drosophila* TRPA1 performs critical sensory functions in chemosensory, photosensory, and thermosensory neurons during both larval and adult stages [17,20-25]. To characterize other neuronal populations that express TRPA1, I examined its expression in *trpA1* ^*GAL4*^, which was generated by a homologous recombination that introduced the *GAL4* gene at the translation initiation codon site for TRPA1 [17]. To examine the spatial distribution of the *trpA1*-*GAL4* reporter in the brain, I introduced a *UAS*-*lacZ* (detected by β-gal antibody) transgene into the *trpA1* ^*GAL4*^/+ background, and then used TH antibody to label dopaminergic neurons as relative landmarks. I observed that β-GAL expression was not collocated with dopaminergic neuron markers in larval brains, and therefore, TRPA1 expression in the larval stage is similar to that described in a previous report (Figure 1A–C) [21]. In addition, I observed a previously undescribed cluster of cells composed of ~30 *trpA1* neurons (n = 10, 31.8 ± 0.60) in the adult posterior dorsal protocerebrum (PDP cluster) of the adult, which did not overlap with insulin like peptide 2 (dILP2) expression (Figure S1). In addition, 1 neuron located above the antennal lobe projected symmetrically to the fan-shaped body (FB; Figure 1D, E). I also observed that anterior cell (AC) neurons, which were previously reported to play an important role in adult temperature sensation, projected to DP (Figure 1D, E) [25]. Moreover, *trpA1* ^*GAL4*^ reporter expression was similar between wild-type and *trpA1*-deficient animals, indicating that *trpA1* is not necessary for neuronal survival.

Heavy synaptic arborization was detected in the DP and subesophageal ganglion (SOG) by combining a *trpA1* ^*GAL4*^ driver and a membrane-tethered GFP (*UAS*-*mCD8*::*GFP*), while *UAS*-*mCD8*::*GFP* by itself did not label adult brain cells (Figures S2 and S3C). In these same flies, I examined whether *trpA1* was expressed in circadian neurons. I dissected the brains at ZT 23 after 3 days of LD entrainment, when Per was most abundant and localized to the nucleus, and labeled circadian neurons in these brains with anti-Per. Similar to previous reports, this revealed *trpA1* expression in pacemaker neurons, including 1 LNv, 3 LNds, 2–3 DN1s, 1 DN2, 1 DN3, and 3 LPNs (Figure S3) [13]. These 2 different reporters show the same expression pattern of TRPA1 in circadian neurons, indicating that anti-GFP and anti-β-GAL staining patterns were not non-specific. 

To further characterize the subset of LNv neurons that express *trpA1*, I conducted double-labeling immunohistochemistry studies on *trpA1GAL4*/*UAS*-*nlacZ*::*GFP* flies with anti-β-GAL and anti-PDF. PDF labels 4 l-LNvs and 4 s-LNvs, but not 5^th^ s-LNv [26]. This labeling failed to reveal an overlap in expression between *trpA1* and PDF, which indicates the *trpA1* expressed in the LNv is the 5^th^ s-LNv (Figure S4) [27-29]. Taken together, these results support the hypothesis that *trpA1* functions in central circadian neurons, but outside of the known morning cells.

### Activation of *trpA1* ^+^ neurons by NaChBac decreased morning activity and delayed evening activity following light entrainment at 18°C

**Figure 2:**
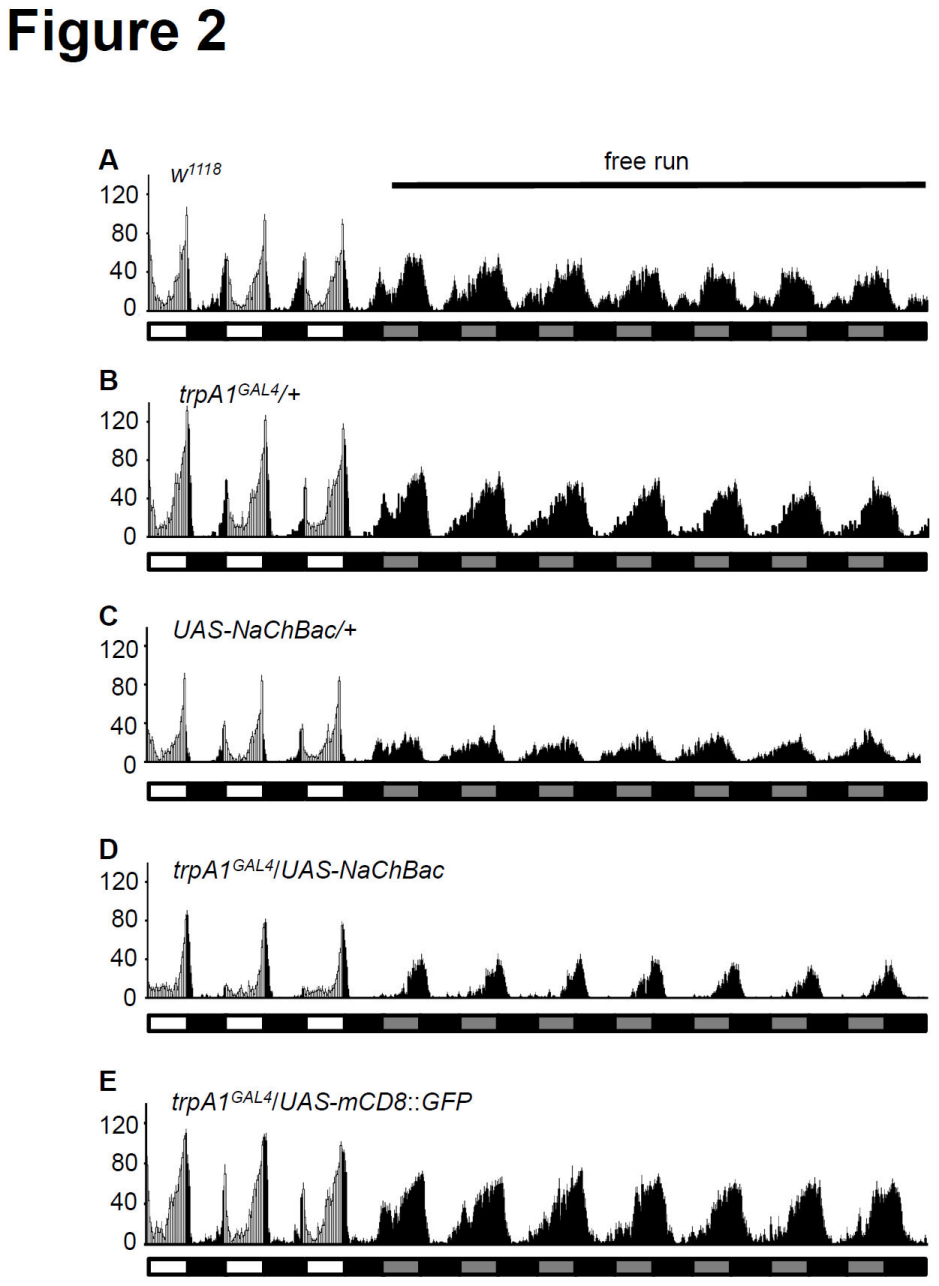
Activation of *trpA1* neurons affects morning peak and evening slope. Averaged activity profiles during light/dark (LD3) and free running at constant dark cycles at 18°C are shown in the following genotypes: (A) *w* ^1118^, (B) *trpA1* ^GAL4^/+, (C) *UAS*-*NaChBac*/+, (D) *UAS*-*NaChBac*/*trpA1* ^*GAL4*^, and (E) UAS-*mCD8*::*GFP*/*trpA1* ^*GAL4*^.

Because *trpA1* is expressed in only a subset of circadian neurons, I asked whether altering the electrical activity of *trpA1* ^+^ neurons could affect circadian behavior during light entrainment. To answer this question, I used voltage-gated sodium channel NaChBac to increase membrane excitability. This halophilic bacterium channel is activated when the membrane potential is near -60 mV, but is reversed when the membrane potential nears 65 mV. Moreover, this channel shows slower activation and inactivation kinetics compared with most voltage-gated sodium channels [30]. I inserted these membrane channels into *trpA1* neurons by crossing *trpA1* ^*GAL4*^ transgenic flies with *UAS*-*NaChBac*. The flies then were loaded at 18°C to evaluate the activating effect of *trpA1* neurons (Figure 2). The temperature threshold for TRPA1 activation, however, is 24°C [31]. Therefore, native TRPA1 remains inactive at 18°C. This approach prevented the TRPA1 channels in the wild-type animals from additionally contributing to the activity of the neurons.

**Figure 3:**
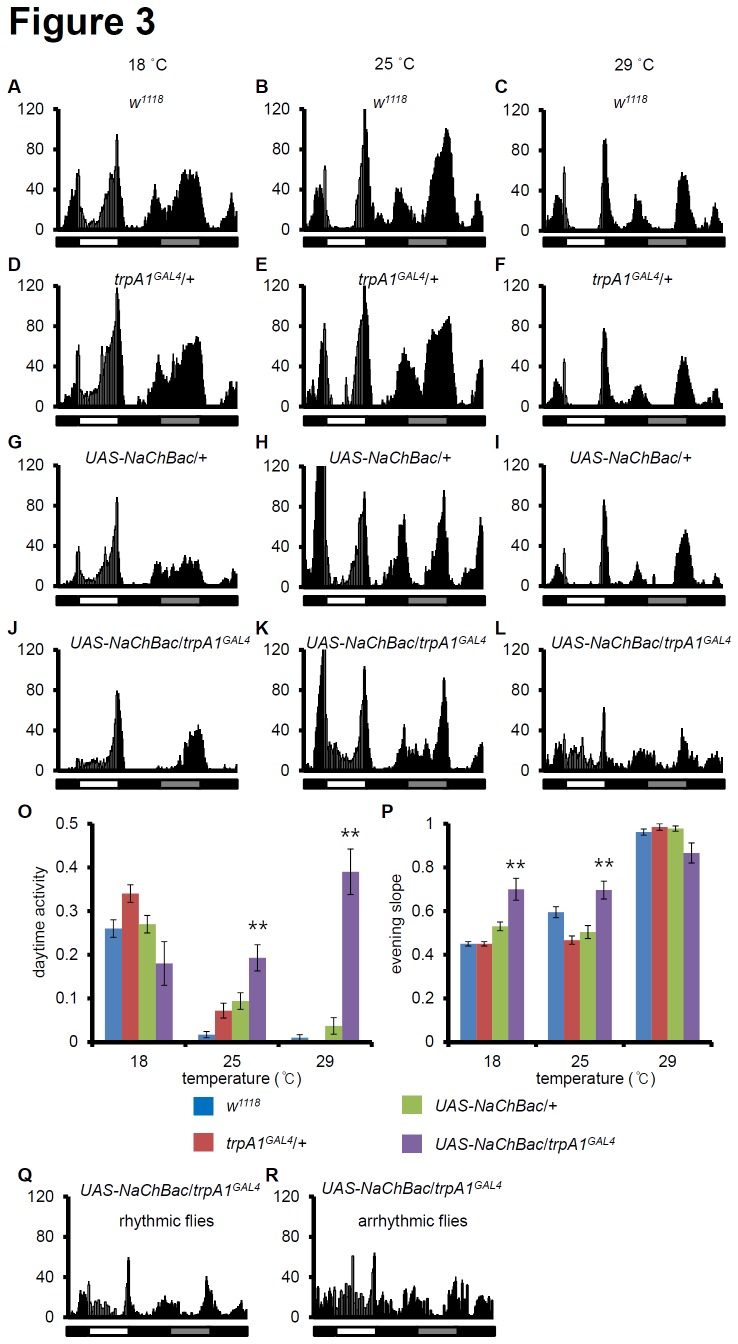
Activation of *trpA1* neurons at different temperatures. Averaged activity profiles during light/dark (LD3) and the first dark cycle (DD1) at 18°/25°/29°C are shown in the following genotypes: (A–C) *w* ^1118^, (D–F) *trpA1* ^GAL4^/+, (G–I) *UAS*-*NaChBac*/+, (J–L) *UAS*-*NaChBac*/*trpA1* ^*GAL4*^. Left, middle, and right panels show the locomotor activity at 18°C, 25°C, and 29°C, respectively. The white, black, and grey bars represent day, night, and subjective day, respectively. (O–P) The daytime activities (O) and the evening slope (P) were computed. For the statistical analysis, the data were analyzed using ANOVA and Scheffe’s post-hoc tests (** *p*-value < 0.01). The *p*-values are relative to wild-type. (Q-R) The locomotor activity of rhythmic (Q) and arrhythmic flies (R) at 29°C.

Next, wild-type and heterozygote control strains were entrained by LD cycles (Figure 2A–C, Table 1). Following entrainment, the flies showed 100% rhythmicity and a normal period between 23.5 h and 24 h (Table 1). Although the MAI and EAI of heterozygote controls were slightly reduced (MAI: *w* ^1118^, 0.72 ± 0.07; *trpA1* ^*GAL4*^/+, 0.39 ± 0.10; *UAS*-*NaChBac*/+, 0.45 ± 0.11, EAI: *w* ^1118^, 0.68 ± 0.05; *trpA1* ^*GAL4*^/+, 0.54 ± 0.04; *UAS*-*NaChBac*/+, 0.54 ± 0.04, not significant), the increasing slopes of evening activity were similar (Figures 2A–C and 3P). The *trpA1GAL4*/*UAS*-*NaChBac* flies, however, unexpectedly showed reduced morning activity even though *trpA1* was not expressed in the morning cells, including the PDF^+^LNvs (Figure 2D, and Figure S4). These animals showed a significant reduction in MAI compared to wild-type (*p* < 0.01) (*w* ^1118^, 0.72 ± 0.07; *trpA1GAL4*/*UAS*-*NaChBac*, 0.14 ± 0.10), but the EAI of *trpA1GAL4*/*UAS*-*NaChBac* flies was comparable to controls (*w* ^1118^, 0.68 ± 0.05; *trpA1GAL4*/*UAS*-*NaChBac*, 0.62 ± 0.10). In contrast, the evening slope of *trpA1GAL4*/*UAS*-*NaChBac* flies was steeper than that for controls (Figures 2A–D, 3P). When *UAS*-*mCD8*::*GFP* was expressed in *trpA1* neurons instead of *UAS*-*NaChBac*, the MAI, EAI, and evening slope were comparable to controls (*trpA1GAL4*/*UAS*-*mCD8*::*GFP*, MAI: 0.38 ± 0.22, EAI: 0.55 ± 0.04, evening slope: 0.42 ± 0.02; Figure 2E).

**Table 1:** Circadian rhythmicity, period, and Fast Fourier Transfer (FFT) values after light/dark (LD) or temperature cycles were analyzed during DD2–DD7 (±SEM).

Genotype	Entrain condition	Entrain Temp (C)	Free run	n	Rhy (%)	Period (h)	FFT
wild-type	LD	18°	18°	32	100	23.77 ± 0.07	0.18 ± 0.01
*trpA1* ^*GAL4*^/+	LD	18°	18°	16	100	23.85 ± 0.02	0.21 ± 0.01
*UAS*-*NaChBac*/+	LD	18°	18°	16	100	23.79 ± 0.05	0.15 ± 0.01
*trpA1GAL4*/*UAS*-*NaChBac*	LD	18°	18°	15	100	23.94 ± 0.08	0.17 ± 0.01
*trpA1GAL4*/*UAS*-*mCD8*:*GFP*	LD	18°	18°	16	100	23.83 ± 0.03	0.20 ± 0.01
wild-type	LD	25°	25°	77	95	23.93 ± 0.03	0.15 ± 0.01
*trpA1* ^*GAL4*^/+	LD	25°	25°	16	100	23.83 ± 0.02	0.21 ± 0.02
*UAS*-*NaChBac*/+	LD	25°	25°	15	100	23.79 ± 0.04	0.15 ± 0.01
*trpA1GAL4*/*UAS*-*NaChBac*	LD	25°	25°	32	100	23.67 ± 0.05	0.14 ± 0.01
wild-type	LD	29°	29°	16	100	23.69 ± 0.06	0.16 ± 0.01
*trpA1* ^*GAL4*^/+	LD	29°	29°	15	100	23.68 ± 0.03	0.14 ± 0.01
*UAS*-*NaChBac*/+	LD	29°	29°	16	100	23.75 ± 0.04	0.15 ± 0.01
*trpA1GAL4*/*UAS*-*NaChBac*	LD	29°	29°	17	65	23.72 ± 0.08	0.07 ± 0.01*
*trpA1* ^*GAL4*^/+	TC	25°/16°	25°	16	100	23.71 ± 0.06	0.20 ± 0.02
*UAS*-*NaChBac*/+	TC	25°/16°	25°	15	93	23.77 ± 0.06	0.16 ± 0.01
*trpA1GAL4*/*UAS*-*NaChBac*	TC	25°/16°	25°	27	96	23.67 ± 0.09	0.14 ± 0.01

The asterisk indicates a significant difference from the wild-type (*p* < 0.05), as determined with an ANOVA and Scheffe’s post-hoc tests.

The morning and evening activity peaks have distinct anatomical requirements. Tissue-specific ablation and rescue experiments were performed for the LNv, which are essential for morning anticipation, and the LNd and DN1, which are required for evening anticipation [7,32]. As previously noted*, trpA1* ^*GAL4*^ is only expressed in small groups of circadian neuron clusters, except PDF^+^LNvs (Figures S3 and S4). Because *trpA1* mutant itself does not affect LD entrainment, these effects at 18°C can not be fully accounted for by TRPA1 activity. Hence, the behavioral effect on the morning activity of *trpA1GAL4*/*UAS*-*NaChBac* flies at lower temperatures suggests that *trpA1* ^+^ neurons should not inhibit morning cells in the low temperature condition. In addition, activation of *trpA1* ^+^ neurons is not involved in evening anticipation, but delayed the timing of the evening activity. These results suggest the neuronal network between evening cells and morning cells is required for fine tuning circadian behavior.

### *trpA1* ^GAL4^/*UAS*-*NaChBac* flies behave differently in a temperature-dependent manner

To further evaluate the effects of *trpA1GAL4*/*UAS*-*NaChBac*, behavioral locomotor activity was monitored at 3 constant temperatures. First, as previously reported, wild-type and controls have sharper morning and evening peaks with reduced locomotor activity during daytime as the temperature increases (Figure 3A–I) [33]. Surprisingly, morning peaks for *trpA1GAL4*/*UAS*-*NaChBac* are present at 25°C and 29°C, although they were nearly absent at 18°C (Figure 3J-L). Furthermore, the morning activity of these flies at 25°C and 29°C was sustained during daytime and the evening peaks were sharply increased. To quantify these effects, the relative locomotor activity was measured during daytime (Figure 3O-P). The daytime activity (mid-day 6-h activity from ZT3 to ZT9 over 12-h daytime activity) of wild-type and controls decreased as the temperature increased (Figure 3O). However, the locomotor activity of *trpA1GAL4*/*UAS*-*NaChBac* during mid-day also greatly increased (Figure 3O). In addition, the evening slopes (the last 1-h activity from ZT11 to ZT12 over the last 3-h light phase) of *trpA1GAL4*/*UAS*-*NaChBac* were significantly sharper at 18°C and 25°C (*p* < 0.01), and the steepness was saturated in controls at 29°C (Figure 3P). These results suggest the morning peaks observed for *trpA1GAL4*/*UAS*-*NaChBac* at different temperatures result from fine-tuning by *trpA1* ^+^ neurons, but the sharp evening peaks and increased daytime activity of *trpA1GAL4*/*UAS*-*NaChBac* were relatively consistent regardless of temperature. Furthermore, 35% of *trpA1GAL4*/*UAS*-*NaChBac* flies showed arrhythmic behavior, whereas the remaining flies showed low rhythmic power at 29°C (Table 1). To quantify differences between rhythmic and arrhythmic flies at 29°C during LD entrainment, the locomotor activity was separated (Figure 3Q, R). Although the daytime activity was comparable (rhythmic: 0.32 ± 0.06, arrhythmic: 0.39 ± 0.10), the evening slope was significantly different (rhythmic: 0.94 ± 0.03, arrhythmic: 0.65 ± 0.13, *p* < 0.01). In conclusion, this finding provides evidence that *trpA1* ^+^ neurons may be important for regulating rhythmicity and circadian power at higher temperatures, but not at low and intermediate temperatures. This interpretation does, however, depend on the assumption that the *trpA1GAL4*/*UAS*-*NaChBac* phenotypes reflect native functions of *trpA1* ^+^ neurons.

### The *trpA1* ^+^ neurons play essential roles in temperature cycle (TC) entrainment through circadian control or clock-independent temperature control of locomotor activity

**Figure 4:**
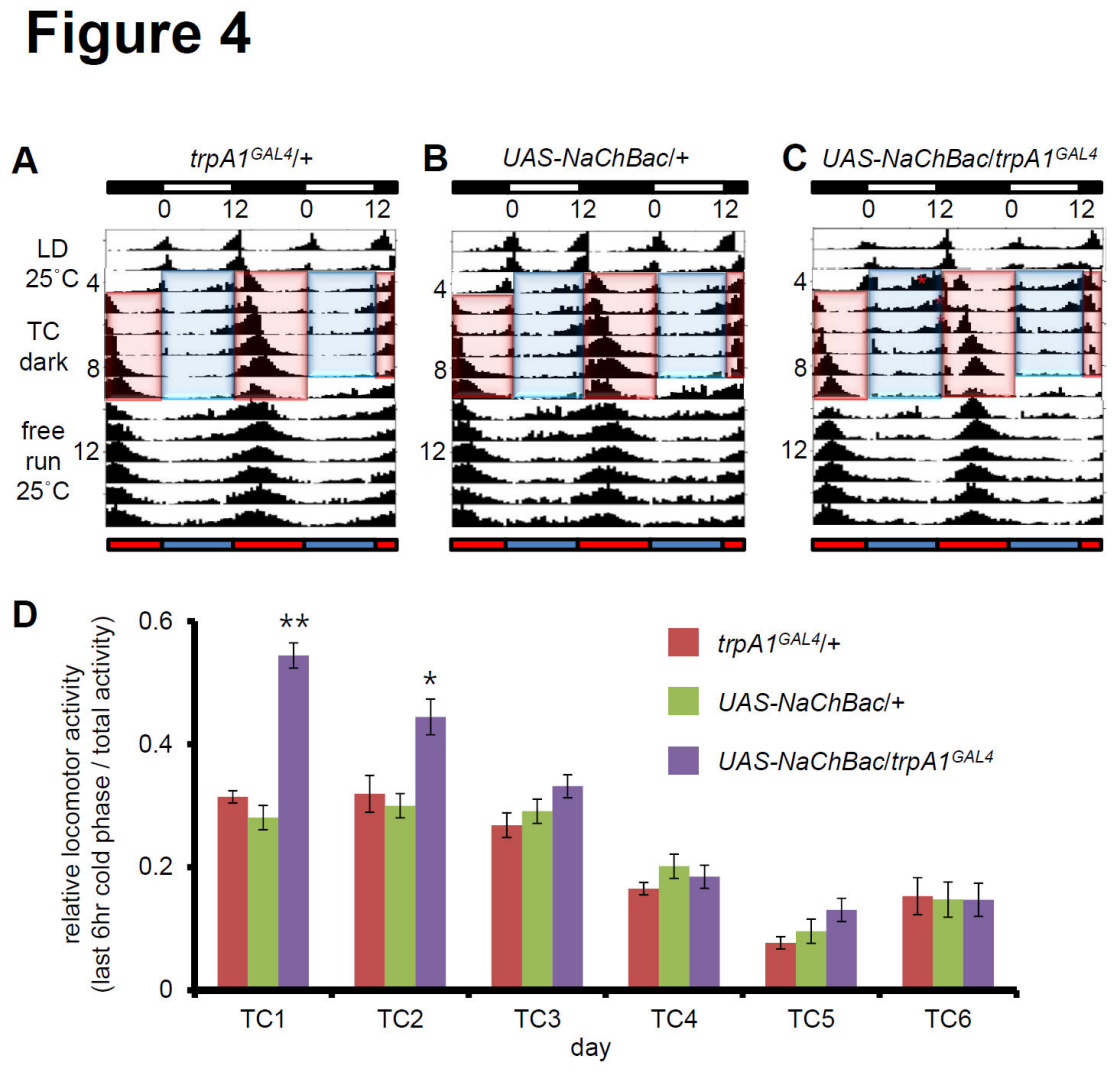
TRPA1 is required for circadian entrainment by temperature. (A–C) Average actograms of flies that were entrained to 12-h:12-h LD cycles for 3 days at 25°C followed by 6 days of DD and 12-h:12-h temperature (25°C/16°C) cycles in which warm and cold phase were in antiphase to the previous light/dark (LD) cycle. After temperature entrainment, the flies were kept at 25°C for free running. Each genotype was denoted on the double plot. Asterisks indicate ectopic peaks that were not detected in controls. Blue and red bars indicate cryophase and thermophase, respectively. (D) Relative locomotor activity during the last 6-h cold phase. The *p*-values are relative to wild-type (** *p*-value < 0.01, * *p*-value < 0.05).

Next, I examined whether the activation of *trpA1* ^+^ neurons can affect temperature entrainment. To evaluate the effect of *trpA1* ^+^ neuronal activation in temperature entrainment, I used TCs (25°C/16°C) in an antiphase paradigm. First, I entrained the flies with an LD cycle at 25°C, and then initiated the first cryophase when the light cycle would have initiated [13,34]. Control flies displayed a robust single peak in the middle of the thermophase, and the single peak persisted for many days during the free running period at 25°C (Figure 4A, B). The robust single peak in controls was much sharper in *trpA1GAL4*/*UAS*-*NaChBac*, reflecting the sharpened evening peaks during LD entrainment (Figures 2D, 3J–L, and 4C). Furthermore, these flies show additional prolonged peaks during cryophase for several days (Figure 4C). The activity peaks during cryophase (the last 6-h cold phase over total activity) were larger than those observed in the middle of the thermophase in *trpA1GAL4*/*UAS*-*NaChBac* (Figure 4D). This finding suggests that the cold phase during TC entrainment should block activation of morning activity in the LD cycle. Nevertheless, the *trpA1GAL4*/*UAS*-*NaChBac* flies consistently exhibited activated warm neurons during the cold phase. Once the morning activity was activated in *trpA1GAL4*/*UAS*-*NaChBac*, the evening activity was decreased (Figure 4C). After day 3, *trpA1GAL4*/*UAS*-*NaChBac* showed locomotor activity that matched the level of controls. Taken together, these findings suggest *trpA1* ^+^ neurons play a role in TC entrainment through circadian control or clock-independent temperature control of locomotor activity.

## Discussion

Temperature entrainment of the central clock depends in part on peripheral sensory neurons [35]. Here, I provide evidence that a subset of central pacemaker neurons expressing TRPA1 upon hyperexcitation also functions in temperature synchronization of circadian rhythms. Recently, we proposed that TRPA1 is functionally important in 2 classes of pacemaker neurons, LPNs and 3 LNds, because TRPA1 was expressed in all 3 LPNs and 2 Cry-negative LNds [13]. In this study, I found that only one s-LNv, which does not express PDF, is *trpA1* ^+^ (Figure S4). Tissue-specific ablation and rescue experiments have shown that PDF^+^LNv neurons are required for morning activity, whereas LNd and DN1 neurons are required for evening activity [7,32]. These findings suggest that the TRPA1 expressing central pacemaker neurons might contribute to evening activity rather than morning activity. However, multiple aspects of my current findings contradict this idea. First, when TRPA1 neurons are activated by *NaChBac*, the morning peaks are strongly suppressed when the experiments were performed at 18°C, and evening peaks were always much sharper than those for controls, regardless of temperature (Figure 3). This suggests that morning activity and evening activity may be controlled in different ways, but they are controlled in a temperature-dependent manner. At cold temperatures (18°C), cold sensing neurons may dominantly suppress morning activity, which explains why constitutively activated TRPA1 neurons cannot activate morning activity at colder temperatures. At warm or hot temperatures (25°C or 29°C), warm sensing neurons may dominantly activate morning activity. For evening activity, heat sensing neurons may dominantly suppress the initiation of evening activity, regardless of constant temperature conditions. This might explain why the *trpA1GAL4*/*UAS*-*NaChBac* flies always showed sharp slopes of evening activity compared to controls. 

Second, daytime activity is highly dependent on the TRPA1 neurons. Indeed, a small peak during daytime activity was noticeable at 18°C (Figure 2D). In addition, morning activity for the *trpA1GAL4*/*UAS*-*NaChBac* flies at 25°C and 29°C was much broader than that for controls (Figure 3K, L). This phenomenon may be explained by sustained morning peak activity during the daytime. In a previous report, we showed that *trpA1* mutants have an effect on TC entrainment, but not LD entrainment. Here, I show the *trpA1GAL4*/*UAS*-*NaChBac* flies have defects in LD entrainment. These new findings suggest that the trpA1+ neurons are activated by pathways independent of TRPA1. In addition, *trpA1* mutants have defects on the period length following TC entrainment, but the *trpA1GAL4*/*UAS*-*NaChBac* flies show normal period in LD and TC except LD at higher temperature such as 29°C (Table 1). Thus, there is a difference between *trpA1* mutants and the *trpA1GAL4*/*UAS*-*NaChBac* flies.

TRPA1 neurons may also contribute to the functions of the central pacemaker neurons because TRPA1 is expressed in 1–3 neurons in each cluster. Furthermore, TRPA1 might function as a temperature sensor and modulate the activity of all the neurons within a cluster. For example, LNds play at least 2 roles for sensing environmental changes. Three Cry^+^LNds can sense blue light and control downstream neurons, which allows other Cry^-^ neurons to be synchronized. TRPA1^+^LNds also might control downstream neurons, which participates in synchronization with temperature changes. Recently, it was shown that dorsally located pacemaker neurons mediate synchronization at relatively high TCs (29°C:20°C), whereas ventral pacemaker neurons mediate synchronization at low TCs (25°C:16°C) [36]. Because *trpA1* is expressed in dorsal neurons and not ventral neurons, and the activation threshold of TRPA1 is 27°C *in vivo* [25], it would be interesting to learn whether the *trpA1* ^*GAL4*^ expression could rescue high TC cycles. In addition, circadian rhythms for temperature preference are mediated by DN2 [37], and 1 of 2 DN2s also expresses TRPA1. These recent reports support the functions of *trpA1* ^+^ neurons described in the current study.

Furthermore, we still do not know how to manipulate most DNs because the sizes and functions of DN1s and DN3s are heterogeneous. The *trpA1* ^*GAL4*^ driver provides a new tool to examine the roles of DN1s and DN3s as a marker. A limitation of my current study is that it does not rule out a possible role for TRPA1 in non-circadian neurons including the PDP clusters. Nevertheless, this study raises the possibility that mammalian thermoTRP channels, including TRPA1, might play similar roles in central pacemaker neurons. It will be interesting to discover other roles for TRPA1 in FB and PDP clusters.

## Supporting Information

Figure S1 **TRPA1 expression does not overlap with dILP2 expression in the pars intercerebralis.** **A**–**F**, Colabelling with anti-dILP2 (red, A and D) and anti-GFP (green, B and E) from UAS-*mCD8*::*GFP*/+;*trpA1* ^GAL4^/+ flies, which shows no overlap between PDP cluster of TRPA1 and insulin-producing cells in the pars intercerebralis. C and F are merged images.
(TIF)

Figure S2 **The undriven *UAS*-*mCD8*::*GFP* brain does not show any GFP signal.** No anti-GFP signal is detected in the absence of *trpA1* ^*GAL4*^ reporter.
(TIF)

Figure S3 **TRPA1 is expressed in pacemaker neurons in the brain.** **A**–**L**, Colabelling with anti-Per (red) and anti-GFP (green) from *UAS*-*mCD8*::*GFP*/+;*trpA1* ^*GAL4*^/+ flies, which shows overlap in some dorsal (**D**–**F**), lateral dorsal (**G**–**I**) and lateral ventral (**J**–**L**) pacemaker neurons. Note the dense synaptic arborization in the dorsal protocerebrum (DP), fan-shaped body (FB), and subesophageal ganglion (SOG). C, F, I, and **L** are merged images.
(TIF)

Figure S4 **TRPA1 is not expressed in PDF^+^ LNvs in the brain.** **A**–**C**, Frontal view of colabelling with anti-PDF (red, **J**) and anti-GFP (green, **K**) from *UAS*-*nlacZ*::*GFP*/+;*trpA1* ^*GAL4*^/+ flies. Note that PDF^+^LNvs do not express TRPA1.
(TIF)

## References

[1] Emery P, Stanewsky R, Helfrich-Förster C, Emery-Le M, Hall JC et al. (2000) *Drosophila* CRY Is a Deep Brain Circadian Photoreceptor. Neuron 26: 493-504. doi:10.1016/S0896-6273(00)81181-2. PubMed: 10839367. 10839367

[2] Glaser FT, Stanewsky R (2005) Temperature Synchronization of the *Drosophila* Circadian Clock. Curr Biol 15: 1352-1363. doi:10.1016/j.cub.2005.06.056. PubMed: 16085487. 16085487

[3] Helfrich-Förster C, Winter C, Hofbauer A, Hall JC, Stanewsky R (2001) The circadian clock of fruit flies is blind after elimination of all known photoreceptors. Neuron 30: 249-261. doi:10.1016/S0896-6273(01)00277-X. PubMed: 11343659. 11343659

[4] Allada R, Chung BY (2010) Circadian organization of behavior and physiology in Drosophila. Annu Rev Physiol 72: 605-624. doi:10.1146/annurev-physiol-021909-135815. PubMed: 20148690. 20148690 PMC2887282

[5] Yoshii T, Heshiki Y, Ibuki-Ishibashi T, Matsumoto A, Tanimura T et al. (2005) Temperature cycles drive Drosophila circadian oscillation in constant light that otherwise induces behavioural arrhythmicity. Eur J Neurosci 22: 1176-1184. doi:10.1111/j.1460-9568.2005.04295.x. PubMed: 16176360. 16176360

[6] Yoshii T, Vanin S, Costa R, Helfrich-Förster C (2009) Synergic entrainment of Drosophila’s circadian clock by light and temperature. J Biol Rhythms 24: 452-464. doi:10.1177/0748730409348551. PubMed: 19926805. 19926805

[7] Grima B, Chélot E, Xia R, Rouyer F (2004) Morning and evening peaks of activity rely on different clock neurons of the Drosophila brain. Nature 431: 869-873. doi:10.1038/nature02935. PubMed: 15483616. 15483616

[8] Stoleru D, Peng Y, Nawathean P, Rosbash M (2005) A resetting signal between Drosophila pacemakers synchronizes morning and evening activity. Nature 438: 238-242. doi:10.1038/nature04192. PubMed: 16281038. 16281038

[9] Murad A, Emery-Le M, Emery P (2007) A Subset of Dorsal Neurons Modulates Circadian Behavior and Light Responses in *Drosophila* . Neuron 53: 689-701. doi:10.1016/j.neuron.2007.01.034. PubMed: 17329209. 17329209 PMC1852515

[10] Rieger D, Wülbeck C, Rouyer F, Helfrich-Förster C (2009) Period gene expression in four neurons is sufficient for rhythmic activity of Drosophila melanogaster under dim light conditions. J Biol Rhythms 24: 271-282. doi:10.1177/0748730409338508. PubMed: 19625729. 19625729

[11] Zhang Y, Liu Y, Bilodeau-Wentworth D, Hardin PE, Emery P (2010) Light and Temperature Control the Contribution of Specific DN1 Neurons to *Drosophila* Circadian. Behav - Current Biology 20: 600-605. doi:10.1016/j.cub.2010.02.044. PMC2862552 20362449

[12] Peschel N, Helfrich-Förster C (2011) Setting the clock–by nature: Circadian rhythm in the fruitfly *Drosophila* *melanogaster* . FEBS Lett 585: 1435-1442. doi:10.1016/j.febslet.2011.02.028. PubMed: 21354415. 21354415

[13] Lee Y, Montell C (2013) Drosophila TRPA1 Functions in Temperature Control of Circadian Rhythm in Pacemaker Neurons. J Neurosci 33: 6716-6725. doi:10.1523/JNEUROSCI.4237-12.2013. PubMed: 23595730. 23595730 PMC3664735

[14] Benito J, Houl JH, Roman GW, Hardin PE (2008) The blue-light photoreceptor CRYPTOCHROME is expressed in a subset of circadian oscillator neurons in the Drosophila CNS. J Biol Rhythms 23: 296-307. doi:10.1177/0748730408318588. PubMed: 18663237. 18663237 PMC2536721

[15] Wolfgang W, Simoni A, Gentile C, Stanewsky R (2013) The Pyrexia transient receptor potential channel mediates circadian clock synchronization to low temperature cycles in Drosophila melanogaster. Proc Biol Sci 280: 20130959 PubMed: 23926145. 23926145 10.1098/rspb.2013.0959 PMC3757961

[16] Lee Y, Lee Y, Lee J, Bang S, Hyun S et al. (2005) Pyrexia is a new thermal transient receptor potential channel endowing tolerance to high temperatures in Drosophila melanogaster. Nat Genet 37: 305-310. doi:10.1038/ng1513. PubMed: 15731759. 15731759

[17] Kim SH, Lee Y, Akitake B, Woodward OM, Guggino WB et al. (2010) Drosophila TRPA1 channel mediates chemical avoidance in gustatory receptor neurons. Proc Natl Acad Sci U S A 107: 8440-8445. doi:10.1073/pnas.1001425107. PubMed: 20404155. 20404155 PMC2889570

[18] Lee Y, Moon SJ, Montell C (2009) Multiple gustatory receptors required for the caffeine response in Drosophila. Proc Natl Acad Sci U S A 106: 4495-4500. doi:10.1073/pnas.0811744106. PubMed: 19246397. 19246397 PMC2657413

[19] Lear BC, Lin J-M, Keath JR, McGill JJ, Raman IM et al. (2005) The Ion Channel Narrow Abdomen Is Critical for Neural Output of the *Drosophila* Circadian Pacemaker. Neuron 48: 965-976. doi:10.1016/j.neuron.2005.10.030. PubMed: 16364900. 16364900

[20] Xiang Y, Yuan Q, Vogt N, Looger LL, Jan LY et al. (2010) Light-avoidance-mediating photoreceptors tile the Drosophila larval body wall. Nature 468: 921-926. doi:10.1038/nature09576. PubMed: 21068723. 21068723 PMC3026603

[21] Rosenzweig M, Brennan KM, Tayler TD, Phelps PO, Patapoutian A et al. (2005) The Drosophila ortholog of vertebrate TRPA1 regulates thermotaxis. Genes Dev 19: 419-424. doi:10.1101/gad.1278205. PubMed: 15681611. 15681611 PMC548941

[22] Kwon Y, Shim H-S, Wang X, Montell C (2008) Control of thermotactic behavior via coupling of a TRP channel to a phospholipase C signaling cascade. Nat Neurosci 11: 871-873. doi:10.1038/nn.2170. PubMed: 18660806. 18660806

[23] Kwon Y, Kim SH, Ronderos DS, Lee Y, Akitake B et al. (2010) *Drosophila* TRPA1 Channel Is Required to Avoid the Naturally Occurring Insect Repellent Citronellal. Curr Biol 20: 1672-1678. doi:10.1016/j.cub.2010.08.016. PubMed: 20797863. 20797863 PMC2946437

[24] Kang K, Pulver SR, Panzano VC, Chang EC, Griffith LC et al. (2010) Analysis of Drosophila TRPA1 reveals an ancient origin for human chemical nociception. Nature 464: 597-600. doi:10.1038/nature08848. PubMed: 20237474. 20237474 PMC2845738

[25] Hamada FN, Rosenzweig M, Kang K, Pulver SR, Ghezzi A et al. (2008) An internal thermal sensor controlling temperature preference in Drosophila. Nature 454: 217-220. doi:10.1038/nature07001. PubMed: 18548007. 18548007 PMC2730888

[26] Renn SC, Park JH, Rosbash M, Hall JC, Taghert PH (1999) A *pdf* Neuropeptide Gene Mutation and Ablation of PDF Neurons Each Cause Severe Abnormalities of Behavioral Circadian Rhythms in *Drosophila* . Cell 99: 791-802. doi:10.1016/S0092-8674(00)81676-1. PubMed: 10619432. 10619432

[27] Helfrich-Förster C (1995) The period clock gene is expressed in central nervous system neurons which also produce a neuropeptide that reveals the projections of circadian pacemaker cells within the brain of Drosophila melanogaster. Proc Natl Acad Sci U S A 92: 612-616. doi:10.1073/pnas.92.2.612. PubMed: 7831339. 7831339 PMC42792

[28] Renn SC, Park JH, Rosbash M, Hall JC, Taghert PH (1999) A< i> pdf</i> Neuropeptide Gene Mutation and Ablation of PDF Neurons Each Cause Severe Abnormalities of Behavioral Circadian Rhythms in< i> Drosophila</i >. Cell 99: 791-802 10.1016/s0092-8674(00)81676-1 10619432

[29] Park JH, Helfrich-Förster C, Lee G, Liu L, Rosbash M et al. (2000) Differential regulation of circadian pacemaker output by separate clock genes in Drosophila. Proc Natl Acad Sci U S A 97: 3608-3613. doi:10.1073/pnas.97.7.3608. PubMed: 10725392. 10725392 PMC16287

[30] Nitabach MN, Wu Y, Sheeba V, Lemon WC, Strumbos J et al. (2006) Electrical hyperexcitation of lateral ventral pacemaker neurons desynchronizes downstream circadian oscillators in the fly circadian circuit and induces multiple behavioral periods. J Neurosci 26: 479-489. doi:10.1523/JNEUROSCI.3915-05.2006. PubMed: 16407545. 16407545 PMC2597197

[31] Viswanath V, Story GM, Peier AM, Petrus MJ, Lee VM et al. (2003) Opposite thermosensor in fruitfly and mouse. Nature 423: 822-823. doi:10.1038/423822a. PubMed: 12815418. 12815418

[32] Stoleru D, Peng Y, Agosto J, Rosbash M (2004) Coupled oscillators control morning and evening locomotor behaviour of Drosophila. Nature 431: 862-868. doi:10.1038/nature02926. PubMed: 15483615. 15483615

[33] Majercak J, Sidote D, Hardin PE, Edery I (1999) How a circadian clock adapts to seasonal decreases in temperature and day length. Neuron 24: 219-230. doi:10.1016/S0896-6273(00)80834-X. PubMed: 10677039. 10677039

[34] Busza A, Murad A, Emery P (2007) Interactions between circadian neurons control temperature synchronization of Drosophila behavior. J Neurosci 27: 10722-10733. doi:10.1523/JNEUROSCI.2479-07.2007. PubMed: 17913906. 17913906 PMC6672815

[35] Sehadova H, Glaser FT, Gentile C, Simoni A, Giesecke A et al. (2009) Temperature Entrainment of *Drosophila*'s Circadian Clock Involves the Gene *nocte* and Signaling from Peripheral Sensory Tissues to the Brain. Neuron 64: 251-266. doi:10.1016/j.neuron.2009.08.026. PubMed: 19874792. 19874792

[36] Gentile C, Sehadova H, Simoni A, Chen C, Stanewsky R (2013) Cryptochrome Antagonizes Synchronization of *Drosophila*’s Circadian Clock to Temperature. Cycles - Current Biology 23: 185-195. doi:10.1016/j.cub.2012.12.023. 23333312

[37] Kaneko H, Head LM, Ling J, Tang X, Liu Y et al. (2012) Circadian rhythm of temperatrue preference and its neural control in *Drosophila* . Current Biology 22: 1851-1857. doi:10.1016/j.cub.2012.08.006. PubMed: 22981774. 22981774 PMC3470760

