# Supplementary figures and images for: Contribution of Drosophila TRPA1-Expressing Neurons to Circadian Locomotor Activity Patterns
Source: PLoS One. 2013 Dec 18;8(12):e85189. doi: 10.1371/journal.pone.0085189 (PMC3867552; doi:10.1371/journal.pone.0085189)

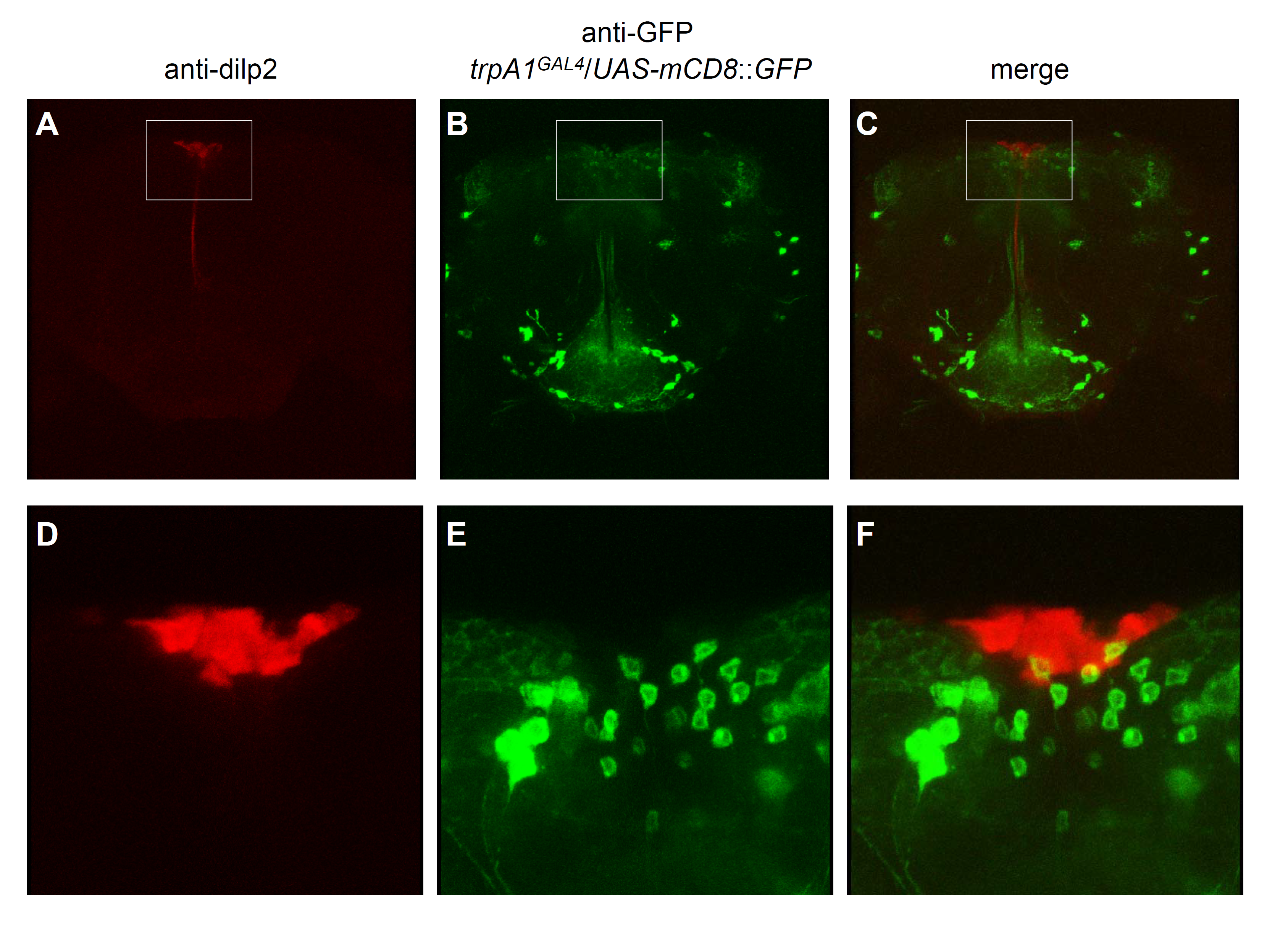

Supplement: Figure S1 — TRPA1 expression does not overlap with dILP2 expression in the pars intercerebralis. A–F, Colabelling with anti-dILP2 (red, A and D) and anti-GFP (green, B and E) from UAS-mCD8::GFP/+;trpA1 GAL4/+ flies, which shows no overlap between PDP cluster of TRPA1 and insulin-producing cells in the pars intercerebralis. C and F are merged images. (TIF) [file pone.0085189.s001.tif]

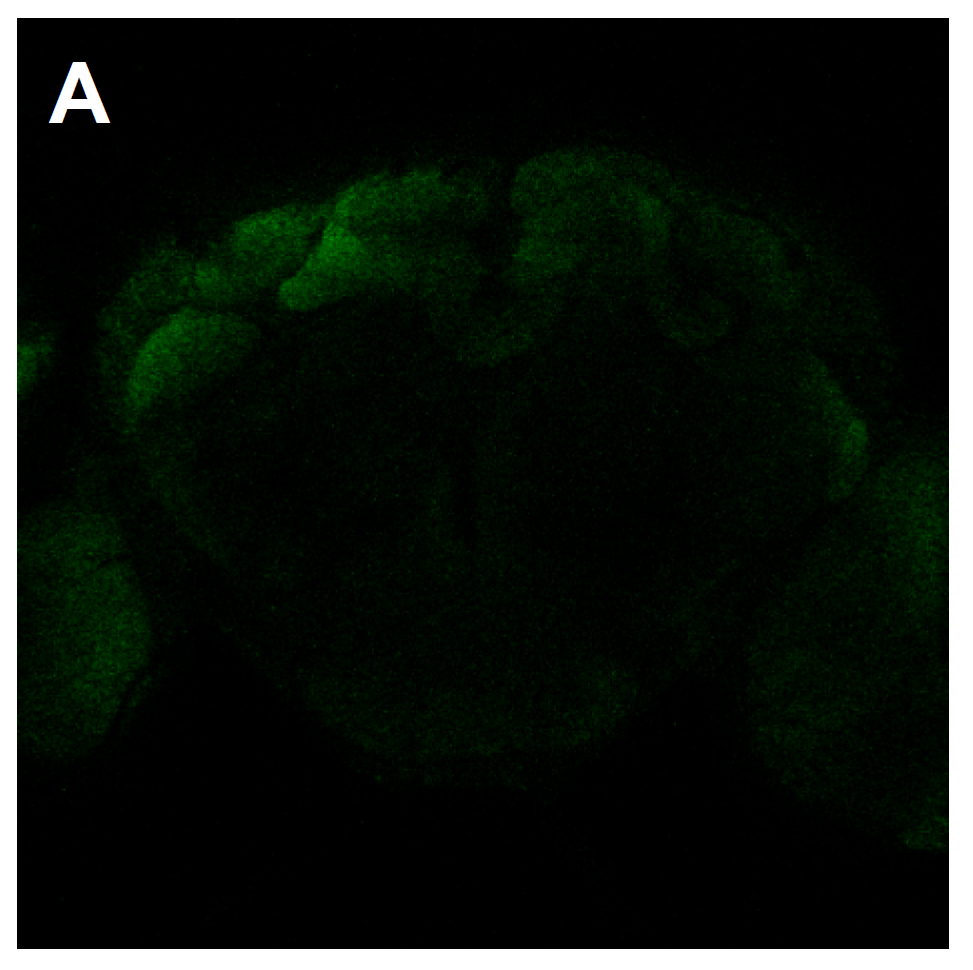

Supplement: Figure S2 — The undriven UAS-mCD8::GFP brain does not show any GFP signal. No anti-GFP signal is detected in the absence of trpA1 GAL4 reporter. (TIF) [file pone.0085189.s002.tif]

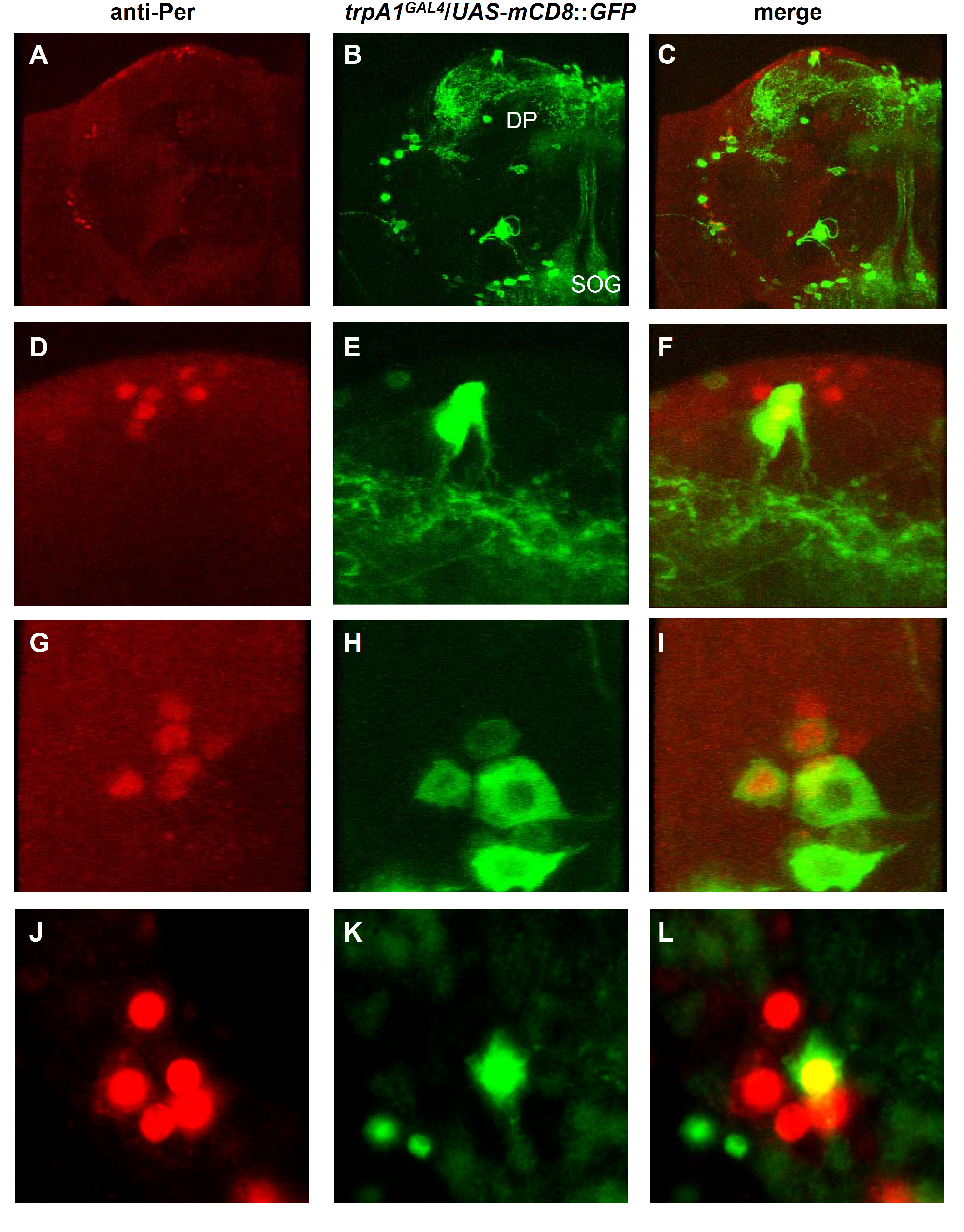

Supplement: Figure S3 — TRPA1 is expressed in pacemaker neurons in the brain. A–L, Colabelling with anti-Per (red) and anti-GFP (green) from UAS-mCD8::GFP/+;trpA1 GAL4/+ flies, which shows overlap in some dorsal (D–F), lateral dorsal (G–I) and lateral ventral (J–L) pacemaker neurons. Note the dense synaptic arborization in the dorsal protocerebrum (DP), fan-shaped body (FB), and subesophageal ganglion (SOG). C, F, I, and L are merged images. (TIF) [file pone.0085189.s003.tif]

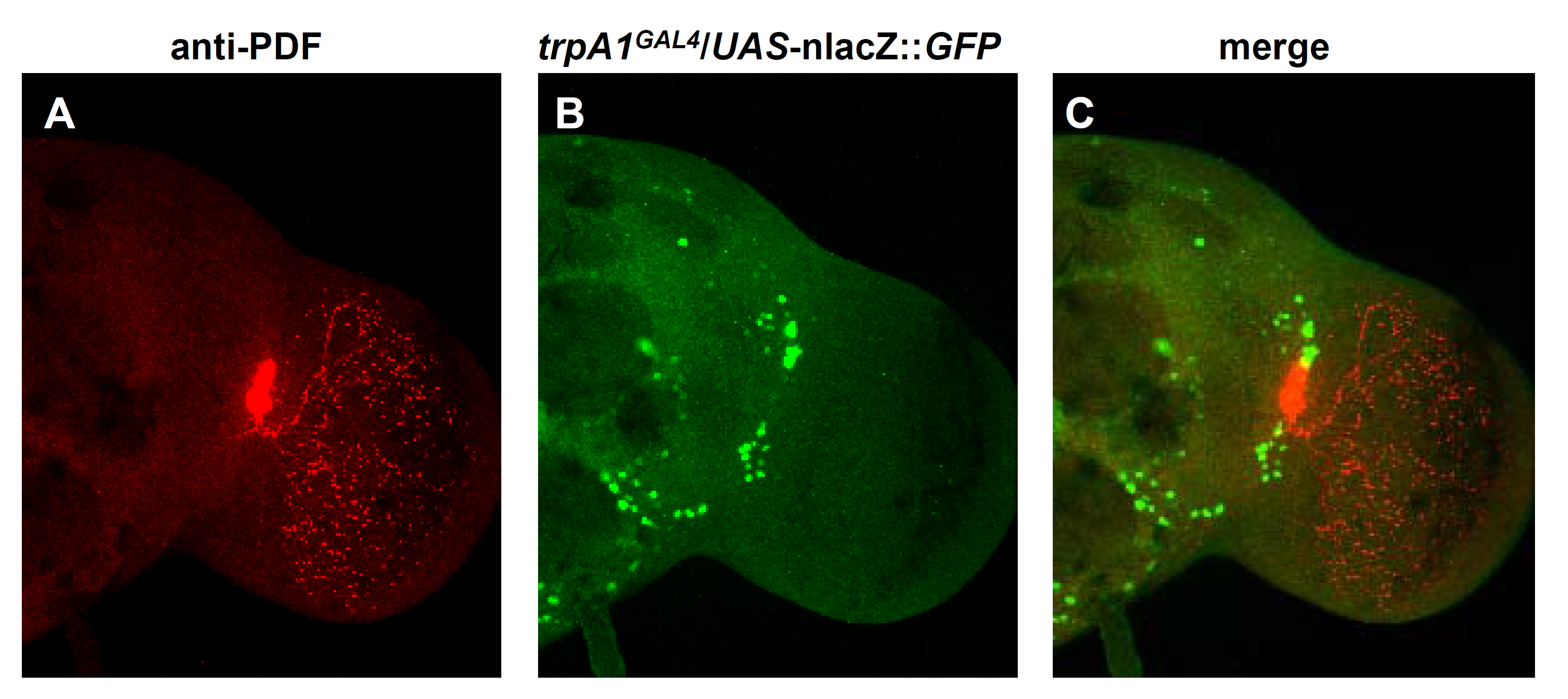

Supplement: Figure S4 — TRPA1 is not expressed in PDF+ LNvs in the brain. A–C, Frontal view of colabelling with anti-PDF (red, J) and anti-GFP (green, K) from UAS-nlacZ::GFP/+;trpA1 GAL4/+ flies. Note that PDF+LNvs do not express TRPA1. (TIF) [file pone.0085189.s004.tif]
